# Accuracy of Predictive Resting-Metabolic-Rate Equations in Chinese Mainland Adults

**DOI:** 10.3390/ijerph16152747

**Published:** 2019-08-01

**Authors:** Jingjing Xue, Shuo Li, Yong Zhang, Ping Hong

**Affiliations:** 1 China Institute of Sport Science, Beijing 100061, China; 2 School of Sport Science, Shanghai University of Sport, Shanghai 200438, China; 3 Tianjin Key Laboratory of Exercise Physiology and Sports Medicine, Tianjin University of Sport, Tianjin 301617, China; 4 Winter Sports Administrative Center, General Administration of Sport of China, Beijing 100044, China

**Keywords:** resting metabolic rate, indirect calorimetry, equations, Chinese mainland adults

## Abstract

Accurate measurement of the resting metabolic rate (RMR) is necessary when we make energy requirements and nutrition suggestions in clinical. However, indirect calorimetry is not always available. The objectives of this study were to make a comparison between RMR measured by indirect calorimetry and RMR predicted by different kinds of equations, and to develop new predictive equations for Chinese mainland adults. In this study, 315 Chinese mainland adults from different provinces all over China were recruited. Subjects underwent half a day of testing, which consisted of anthropometric assessment and RMR measurement. Measured and predicted RMR were compared; new optimal equations for Chinese mainland adults were developed and tested by splitting the subjects into a development and validation group. The measured RMR was in the range of 831–2776 kcal/day (mean 1651 ± 339 kcal/day). Our findings indicated that, except for the Harris–Benedict and Schofield equations, three Chinese equations and two fat-free mass (FFM) modeling equations all significantly underestimated RMR compared to the measured value (all *p* < 0.01). There were no significant differences between predicted and measured RMR using the new equations for females and males. Of the pre-existing equations, Schofield’s is the most suitable for Chinese mainland adults. However, the two new equations developed in this study seem to be more effective for predicting the RMR of Chinese mainland adults, and need to be validated by a larger independent sample with different physiological and anthropometric characteristics.

## 1. Introduction

Resting metabolic rate (RMR) is typically defined as the energy required when we are in a resting condition [1], which is very important because it typically contributes 60–75% of total energy expenditure in humans [2]. RMR is a necessary and important factor when determining daily energy requirements for body weight-loss interventions and body-composition management. 

Indirect calorimetry, which is considered to be the clinical gold-standard measurement of RMR, is a metabolic measurement system that tests oxygen consumption and carbon dioxide production to calculate energy expenditure [3]. However, because of the high cost, the time it requires (30–50 min), and because it is not widely available, the practicality of using indirect calorimetry is limited. Consequently, several equations have been used to predict RMR as a substitute for measured RMR [4], by using some anthropometric indices, such as age, body weight, height, gender, and fat-free mass. We cannot accurately obtain all individuals’ RMR by using one standard prediction equation because each RMR prediction equation has been developed for a specific race, age group, or based on various other physical characteristics [5], which all differ from the characteristics of other subjects. 

Comparisons have been made between various equations and measured RMR in several studies. Most of these studies have shown that overestimations or underestimations are found when the equations are used in different populations [6]. Consequently, in order to improve the equations’ accuracy rate, other equations have been developed according to different populations [7,8,9,10]. We also found that the commonly used predictive equations are based on Western populations, and very few have been developed from Asian populations [11]. There are very few studies on the accuracy rate of different RMR equations applied to Chinese people, although a few equations have been developed from Chinese populations in the last twenty years. For example, the equations of Liu et al. [12], Yang et al. [13], and Singapore [14] were developed in Chinese adults, but their accuracy and applicability need to be examined further. 

In the United States, Australia, and several European countries, the Harris–Benedict equation [15] and the Schofield equation [16] are the two most routinely used equations. Though some researchers have shown that equations from Western populations are not suitable for Asians [17,18], whether these equations are suitable for Chinese mainland people, and their accuracy still needs to be further validated. As we all know, fat-free mass (FFM) is one key factor that affects RMR. The strong association of FFM with RMR has spurred researchers to develop equations based on body-composition data [19]. However, body-composition modeling equations are not popularly used and have not been developed in mainland China.

Therefore, the first objective of this study was to compare measured RMR with predicted RMR by using (a) internationally used equations, (b) Chinese-specific predictive equations, and (c) FFM modeling equations; the second goal was to develop new equations, if possible, body composition modeling equations were preferred from our data applicable for Chinese mainland adult populations without age limits and body-mass-index (BMI) limits.

## 2. Materials and Methods

### 2.1. Subjects

Three hundred and fifteen healthy Chinese subjects from different provinces of mainland China, aged 18–67 years (BMI: 16.7–38.2 kg/m^2^; male: *n* = 127, female: *n* = 188), volunteered to take part in this study. Healthy adults (age range: 18–70 years) with no disease history were eligible for this study. Subjects who had diseases that affect body energy metabolism, such as asthma, chronic obstructive pulmonary disease, hyperthyroidism, diabetes mellitus, and hypertension were excluded from this study. 

All participants were asked to sign an informed consent form. Participants were reimbursed 300 RMB for their participation. The guidelines in the Declaration of Helsinki were followed in this study, and all procedures involving human subjects were approved by the China Institute of Sport Science Committee (Ethical code: CISSIRD-201604).

### 2.2. Measurements

All subjects were asked to take off shoes and heavy clothing before any measurement; we took all measurements between 08:00 and 10:00 to avoid diurnal variation differences. Before the resting-metabolic-rate measurements, all participants underwent anthropometric measurements and demographic data collection.

#### 2.2.1. Anthropometric Measurements

The same trained tester performed all anthropometric measurements including body height, weight, and composition. A bioelectrical impedance analysis composition analyzer (Inbody 770, Biospace Corp., Seoul, Korea) was used to evaluate body composition, which was validated as having a high correlation with dual-energy X-ray absorptiometry (DXA) in estimating body-fat percentage in Chinese subjects [20]. We recorded the mean height and weight results, which were taken twice to the nearest 0.1 kg and 0.1 cm (Height and Weight Scale, Changzhou, China). The BMI = weight (kg)/height (m) squared formula was used to calculate the BMI of the subjects.

#### 2.2.2. RMR Measurement and Prediction

Resting metabolic rate was measured with a ventilated mask by one trained tester in the morning using indirect calorimetry (Cortex Metamax 3B-R2 metabolic system, Leipzig, Germany). Before measurement, subjects were told to have 6–8 h sleep, to not undertake intense physical activity in the previous 24 h, and to fast overnight before arriving at the laboratory. Calibration of flow and gas analyzers was done before each measurement according to the manufacturer’s instructions. Flow calibration was performed, and gas analyzers were calibrated with a standard gas mixture (15% O_2_, 5% CO_2_) and dried atmospheric air (20.93% O_2_, 0.03% CO_2_). Measurement was carried out in a quiet room with dim lighting and controlled environment temperature (22–25 °C) and humidity (40–50%). Subjects were asked to lie in a supine position, and stay quiet and awake during the measurement. The volume of oxygen consumption and carbon dioxide production was measured for a period of 30 min, and then the RMR was calculated by using the VO_2_ and VCO_2_ of the steady periods for about 20 min according to the Weir formula [21]. 

In addition to the RMR measurement using indirect calorimetry, RMR was predicted by the internationally used equations of Harris–Benedict (1919) and Schofield (1985); the Chinese equations of Liu (1995), Yang (2010) and the Singapore equation (2016); and the FFM modeling equations of Cunningham (1980) [22] and Wang et al. (2000) (see Table 1) [23]. Equations developed for particular populations were excluded. The Harris–Benedict and Schofield equations were selected since they are commonly used internationally; the Liu, Yang, and Singapore equations were included since they were developed from Chinese populations; the Cunningham and Wang equations, which were derived from meta-analysis were included because of their use of the relationship of RMR with body-composition data.

Finally, Present 1 and Present 2 were developed for Chinese mainland adults (Table 1). To test the internal validity of the equations, and to control for type I error rate, the sample was split into a 75% development subsample (male: *n* = 95, female: *n* = 140, age: 20–67 years, BMI: 16.7–38.1 kg/m^2^) and a 25% validation subsample (male: *n* = 32, female: *n* = 48, age: 19–61 years, BMI: 16.9–32.2 kg/m^2^).

#### 2.2.3. Statistics

All results are shown as mean ± standard deviation. The SPSS statistical package (version17, SPSS Inc., Chicago, IL, USA) was used to analyze the data. Comparisons of differences at the group level were done between the calculated RMR from the equations and the measured RMR via indirect calorimetry by using paired t-tests. The average percentage bias between predicted and measured RMR was used to estimate accuracy at the group level. The Pearson correlation coefficient (*r*) was used to examine the relationship between predicted RMR and measured RMR. Bland–Altman plots [24] were created using Med-Calc for the males and females of each equation to provide the limits of agreement, the bias between predicted RMR and measured RMR. The limits of agreement were defined as the mean difference ± 1.98 SD. The accuracy of the predictive equations at the individual level was defined as the percentage of predicted RMR that was within ±10% of the measured RMR; overpredictions were considered to be ≥10%, and underpredictions were ≤−10% [25]. The new prediction equations (Chinese mainland equations) were developed by using multiple stepwise regression analysis to estimate RMR based on demographic and anthropometric data. The new prediction equations were generated from the development group and were also tested from the validation group by using the Bland–Altman method. A value of *p* < 0.05 was defined as statistically significant.

## 3. Results

### 3.1. Subject Characteristics

Table 2 shows the physical characteristics of the subjects. All subjects in this study, between 18 and 67 years old, were from different regions of mainland China. The average age was 35.3 ± 12.8 years; average BMI was 23.1 ± 3.2 kg/m^2^ (16.7–38.2 kg/m^2^).

### 3.2. Measured RMR vs. Predicted RMR

Comparisons of the predicted RMR from all equations with the measured RMR are presented in Table 3. The mean measured RMR derived from indirect calorimetry was 1651 ± 339 kcal/day in total (831–2776 kcal/day); the mean of the measured RMR was 1934 ± 286 kcal/day in men and 1460 ± 215 kcal/day in women. Paired sample t-tests demonstrated that all equations except for the Schofield equation significantly underestimated the measured RMR for males, there were no significant differences between Schofield’s predicted RMR and measured RMR; the Harris–Benedict equation overestimated measured RMR for females and all other pre-existing equations significantly underestimated the measured RMR for females. There were no significant differences between measured and predicted values for males and females by the two new equations (Table 3). 

### 3.3. Correlation between Measured RMR and Predicted RMR

Measured RMR via indirect calorimetry for all participants had a significant positive correlation with predicted RMR, with the results as follows: the Harris–Benedict equation (*r =* 0.244, *p* < 0.01), Schofield equation (*r* = 0.758, *p* < 0.01), Liu equation (*r* = 0.727, *p* < 0.01), Yang equation (*r* = 0.725, *p* < 0.01) and the Singapore equation (*r* = 0.756, *p* < 0.01); the Cunningham equation (*r* = 0.776, *p* < 0.01), and Wang equation (*r* = 0.776, *p* < 0.01); and Present equation 1 (*r* = 0.846, *p* < 0.01); Present equation 2 (*r* = 0.819, *p* < 0.01) (Table 3).

### 3.4. Agreement between Measured RMR and Predicted RMR

**Figure 1:**
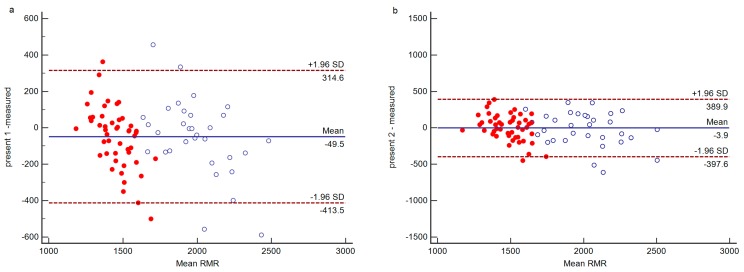
Bland–Altman plot for RMR between present equations ((**a**) Present 1, (**b**) Present 2) and indirect calorimetry (IC) data (*n* = 80). Solid horizontal line presents mean difference between the two methods in kcal/day. Dashed lines depict 95% limits of agreement (mean difference ± 1.96 SD) in kcal/day. Solid circle: female, hollow circle: male.

The mean difference, limits of agreement, and 95% confidence interval for the bias between measured RMR and predicted RMR are presented in Table 4. For males and females, in the pre-existing equations, the lowest mean difference and bias percent difference at the group level between measured RMR and predicted RMR were found in the Schofield prediction equation with a mean difference of 28.2 and 35.9 kcal/day, respectively, −0.34% bias difference in total, and 95% confidence interval for the bias from −16.4 to 72.9 kcal/day and from 7.4 to 64.4 kcal/day, respectively. As shown in Table 4, the range of accuracy rates at the individual level between equations varied from 17.5 to 70%. Among the pre-existing equations, the Schofield equation provided the highest percentage of accurate prediction with 59.1%, 18.7% overprediction, and 22.2% underprediction. Meanwhile, we found that two Chinese equations (Liu, 76.2% and Singapore, 80.3%) and the Wang equation (80.6%) most underpredicted RMR; the Harris–Benedict, Yang, and Cunningham equations provided accurate prediction at a rate of 37.1%, 37.1%, and 45.1%, respectively, at the individual level. However, the two newly developed equations were the most accurate since there were no significant differences between measured and predicted values for males and females. Present 1 showed the highest prediction accuracy with 70% at the individual level, and Present 2 showed the lowest differences and bias at the group level compared to the pre-existing equations (Table 4; Figure 1). Bland–Altman analyses revealed good agreement for all equations by the fact that 94–96% of the data points fell within two standard deviations.

## 4. Discussion

Hundreds of studies suggest that one equation cannot be accurately used for subjects whose age, sex, race, and physical status is different to those used to formulate the original equation [26]. The purpose of this study was to determine the most accurate equation for Chinese mainland adults without age and BMI limits, and to possibly develop new prediction equations applicable to Chinese mainland adults.

The data suggested that most equations, except for the Schofield equation, could not accurately predict RMR in this study group. Previous published studies have indicated that, whatever the race of the subjects, most equations overestimate the RMR of both Caucasian and Asian populations [11,27,28]. However, for both males and females in this study, a significant underestimation of RMR was observed when using all pre-existing Chinese equations, including those of Liu, Yang, and Singapore, which differed from previous studies [13,14,17,18,29] that found these Chinese equations accurately predicted RMR compared to the measured RMR. There are studies that have showed that the Liu equation could predict RMR in Chinese subjects more accurately than others [17,29], but this equation underpredicted in our study subjects. Liu’s equation was developed from a Chinese Taipei population aged 20–78 years, and excluded underweight and obese populations [12] while the database for Yang’s equation came from the southern areas of China [13]. The Singapore equation came from a population of 223 healthy Chinese Singaporean adults, including overweight and obese subjects [14]. Although the pre-existing equations mentioned above were developed from Chinese populations, we found that about 90% of subjects included in these studies were descendants of populations from the southern areas of China. They were all different to our subjects, who came from the northern, middle, and southern areas of the Chinese mainland, and had a wide age range and BMI range. The reasons why differences existed may be due to the subjects’ physical characteristics, living environments, climatic factors, physical activity levels, and measurement errors. Hence, our results suggests that these three pre-existing Chinese predictive equations (Liu, Yang, and Singapore), which were developed from Chinese Taipei, southern Chinese, and Singaporean Chinese population databases, are not suitable for predicting the RMR of Chinese mainland populations.

Our data showed that the Harris–Benedict equation significantly overestimated the RMR of females at the group level, but significantly underestimated the RMR of males at the group level; accuracy at the individual level was low (37.1%) in total. The Harris–Benedict equations were developed from Caucasian subjects only, so overestimation and underestimation is to be expected [7,15]. The Schofield equation has been validated in many different studies, and it is one of the most widely used equations. The Schofield equation was also the most accurate equation (59.1% accuracy rate and –0.34% bias percentage) among the equations validated in this study for predicting RMR in Chinese mainland adults aged 18–67 years for males and females. There are 7173 RMR data entries in the Schofield database, almost half of these are occupied by data for Italian subjects. These subjects were measured by indirect closed-circuit calorimetry, and were thus more likely to show higher basal metabolic rate (BMR) values compared to open-circuit calorimetry. Though the subjects in this study were racially different from Schofield’s subjects, there was no significant difference between the mean predicted RMR and measured RMR for males. The equation also produced the smallest mean difference (all participants) because our RMR measurement method was closed-circuit calorimetry. 

It has been reported in previous studies that the RMR value was mainly influenced by fat-free mass [7,23,30,31]. It is assumed that a higher RMR is due to a greater proportion of muscle to total body weight [32]. However, in our study two FFM equations based on meta-analysis underpredicted RMR. It is supposed that FFM consists of multiple organs and tissue with different metabolic rates, including the brain, liver, heart, and kidneys, and these account for 60–70% of RMR in adults, whereas their combined weight is <6% of total body weight [33]. Almost three-fifths of the subjects in our study (182 of 315; 57.8%) were young subjects who were leading physically active lives, the other subjects except young who volunteered to take part in the study were more likely to be active than sedentary, they may have more active multiple organs and tissue. Therefore, we hypothesized that Chinese mainland subjects in this study may have a higher metabolic rate and distinctive scaling relationships, which might help to explain why these two FFM equations underestimated RMR. Several studies have concluded that Asians have a lower RMR than Caucasians due to the different races [29,34]; however, many studies have concluded that there are no differences between the RMR of Asians and Europeans [35,36,37]. The studies of Boer and Lawrence indicated that the RMR in Asians per FFM kilogram was higher than that of Caucasians [38,39]; therefore, we speculate that the RMR of Chinese populations is not lower than non-Asians, but even higher for both females and males, given that absolute FFM was not less than that of non-Asians. Thus, these findings suggest that these two predictive FFM equations may not be appropriate for Chinese populations.

Hence, the current study developed two kinds of Chinese-specific equations, one of which was based on easily accessible variables and one based on body-composition variables. We tested the internal validity of the newly derived equations using validation groups, and found that our newly derived equations showed no significant differences with measured RMR. Additionally, they displayed the smallest mean differences and bias at the group level and showed a high accuracy rate at the individual level compared to the other equations.

## 5. Conclusions

We found that the Harris–Benedict and the two FFM modeling equations may not be appropriate for predicting the RMR in Chinese mainland adults. The equations of Liu, Yang, and Singapore significantly underestimated the RMR of subjects in this study. Of all the pre-existing equations we tested, Schofield’s equation proved to be the most appropriate for predicting the RMR of Chinese mainland adults; however, new equations developed in this study seem to perform better than any of the pre-existing equations. Taken together, we conclude that we can use the new RMR equations in cases where RMR cannot be measured by direct methods. In subsequent studies, the equations developed in this study should be tested and validated with a larger independent sample with different physiological and anthropometric characteristics. 

## Figures and Tables

**Table 1:** Equations for resting metabolic rate (RMR). Note: FFM, fat-free mass.

Equation Source	Population	Equation	Units
Harris–Benedict (1919)	Males	66 + (13.7 × weight kg) + (5 × height cm) − (6.8 × age years)	kcal/day
	Females	655 + (9.5 × weight kg) + (1.9 × height cm) + (4.7 × age years)	kcal/day
Schofield (1985)	Males (≤30 years)	15.057 × weight kg + 692.2	kcal/day
	Females (≤30 years)	14.818 × weight kg + 486.6	kcal/day
	Males (30–60 years)	11.472 × weight kg + 873.1	kcal/day
	Females (30–60 years)	8.126 × weight kg + 845.6	kcal/day
Liu (1995)	Males	(13.88 × weight kg) + (4.16 × height cm) − (3.43 × age years)	kcal/day
	Females	(13.88 × weight kg) + (4.16 × height cm) − (3.43 × age years) − 112.4	kcal/day
Yang (2010)	Males	277 + 89 × weight kg + 600	kj/day
	Females	277 + 89 × weight kg	kj/day
Singapore (2016)	Males	52.6 × weight kg + 2788	kj/day
	Females	52.6 × weight kg + 1960	kj/day
Cunningham (1980)	All	21.6 × FFM kg + 501.6	kcal/day
Wang (2000)	All	24.6 × FFM kg + 175	kcal/day
Present 1	All	(13.9 × weight kg) + (247 × gender) − (5.39 × age years) + 855 (female = 0, male = 1; R^2^ = 0.606)	kcal/day
Present 2	All	(26.535 × FFM kg) − (5.06 × age years) + 602.1(R^2^ = 0.607)	kcal/day

**Table 2:** Demographic, anthropometric, and body-composition variables of subjects (*n* = 315).

	Male (*n* = 127)	Female (*n* = 188)	Total (*n* = 315)
Age (years)	32.1 ± 11.8	37.5 ± 13.1	35.3 ± 12.8
Height (cm)	173 ± 6	160 ± 5	165 ± 8
Weight (kg)	71.7 ± 9.9	57.4 ± 8.1	63.1 ± 11.3
BMI (kg/m^2^)	23.9 ± 2.7	22.4 ± 3.3	23.1 ± 3.2
Waist hip ratio	0.86 ± 0.17	0.80 ± 0.10	0.82 ± 0.14
Fat-free mass (kg)	56.0 ± 6.5	39.7 ± 3.9	46.2 ± 9.5
Percentage of body fat (%)	21.5 ± 5.7	30.2 ± 6.5	26.7 ± 7.5
Underweight (%)	0	4.60	4.60
Normal weight (%)	17.9	32.9	50.8
Overweight (%)	18.1	18.1	36.2
Obese (%)	4.24	4.26	8.50
Southern region of China (%)	4.76	12.1	16.8
Central region of China (%)	12.7	15.9	28.6
Northern region of China (%)	22.9	31.8	54.6

Note: BMI, body mass index.

**Table 3:** Comparison of RMR values from predictive equations and indirect calorimetry.

Variable	Male (kcal/day)		Female (kcal/day)		Total (kcal/day)	
	Mean ± SD	Pearson’s Correlation Coefficient	Mean ± SD	Pearson’s Correlation Coefficient	Mean ± SD	Pearson’s Correlation Coefficient
Measured RMR (*n* = 315)	1934 ± 286		1460 ± 215		1651 ± 339	
Predicted RMR from						
Harris–Benedict	1665 ± 263 **	0.502 **	1689 ± 115 **	0.199 **	1678 ± 189	0.244 **
Schofield	1906 ± 206	0.507 **	1424 ± 76 *	0.397 **	1619 ± 277 **	0.758 **
Liu	1584 ± 228 **	0.501 **	1216 ± 127 **	0.406 **	1364 ± 251 **	0.727 **
Yang	1723 ± 249 **	0.528 **	1286 ± 173 **	0.365 **	1462 ± 298 **	0.725 **
Singapore	1561 ± 147 **	0.528 **	1190 ± 102 **	0.365 **	1339 ± 220 **	0.756 **
Cunningham	1712 ± 142 **	0.574 **	1358 ± 84.9 **	0.404 **	1500 ± 339 **	0.776 **
Wang	1553 ± 162 **	0.574 **	1151 ± 96.8 **	0.404 **	1312 ± 235 **	0.776 **
Measured RMR (*n* = 80)	2021 ± 287		1478 ± 177		1695 ± 351	
Predicted RMR from						
Present 1	1971 ± 190	0.665 **	1429 ± 96.3	0.361 **	1646 ± 302	0.846 **
Present 2	2005 ± 228	0.608 **	1486 ± 118	0.316 **	1693 ± 307	0.819 **

Significant difference between measured RMR and predicted RMR at * *p* < 0.05, ** *p* < 0.01.

**Table 4:** Agreement between measured RMR and predicted RMR.

Prediction Equations	Mean Difference ± SD (kcal/day)		Limits of Agreement (Mean Difference ± 1.96 SD) (kcal/day)		95% Confidence Interval for Bias (kcal/day)		Accuracy ^a^ (%)	Over-Predicted ^b^ (%)	Under-Predicted ^c^ (%)	Bias (%)
	Male	Female	Male	Female	Male	Female	Total	Total	Total	Total
Harris–Benedict	269 ± 275	−227 ± 223	−269 and 807	−664 and 210	221 to 317	−259 to −195	37.1	37.8	25.1	5.47
Schofield	28.2 ± 254	35.9 ± 199	−470 and 527	−352 and 424	−16.4 to 72.9	7.40 to 64.4	59.1	18.7	22.2	−0.34
Liu	351 ± 262	244 ± 201	−162 and 864	−149 and 638	305 to 397	216 to 273	20.6	3.18	76.2	−16.1
Yang	211 ± 262	174 ± 222	−303 and 726	−261 and 608	165 to 258	142 to 206	37.1	8.25	54.6	−10.3
Singapore	374 ± 243	271 ± 202	−103 and 851	−125 and 666	331 to 416	242 to 300	17.5	2.22	80.3	−17.5
Cunningham	224 ± 236	102 ± 197	−239 and 687	−284 and 488	182 to 267	73.4 to 130	45.1	9.5	45.4	−7.2
Wang	383 ± 235	310 ± 197	−78.6 and 844	−77.1 and 696	341 to 424	281 to 338	18.1	1.30	80.6	−19.4
Present 1	50.1 ± 215	48.9 ± 169	−371 and 471	−281 and 379	−27.4 to 128	−0.06 to 97.8	70.0	10.0	20.0	−1.9
Present 2	16.0 ± 234	−8.30 ± 179	−443 and 475	−360 and 343	−68.4 to 110	−60.3 to 43.8	62.5	21.2	16.3	1.1

^a^ Percentage of subjects predicted by this predictive equation within ±10% of the measured value. ^b^ Percentage of subjects predicted by this predictive equation ≥10% of the measured value. ^c^ Percentage of subjects predicted by this predictive equation ≤−10% of the measured value.
